# Selective loss of ATP carriers in favour of SLC25A43 orthologues in metamonad mitochondria adapted to anaerobiosis

**DOI:** 10.1098/rsob.240202

**Published:** 2025-08-13

**Authors:** Natalia Janowicz, Vít Dohnálek, Justyna Zítek, Priscila Peña-Diaz, Eva Pyrihová, Martin S. King, Michaela Husová, Vojtěch Žárský, Edmund Kunji, Alena Zikova, Vladimír Hampl, Pavel Dolezal

**Affiliations:** ^1^ Department of Parasitology, BIOCEV, Charles University Faculty of Science, Prague, Czech Republic; ^2^ Department of Chemistry, Bioscience Environmental Engineering, University of Stavanger, Stavanger, Norway; ^3^ MRC Mitochondrial Biology Unit, Cambridge Biomedical Campus, University of Cambridge, Cambridge, UK; ^4^ Institute of Parasitology, Biology Centre of the Czech Academy of Sciences, České Budějovice, Czech Republic; ^5^ Department of Biology and Ecology, University of Ostrava, Ostrava, Czech Republic; ^6^ Department of Botany, University of British Columbia, Vancouver, British Columbia, Canada; ^7^ Biology Centre, Academy of Sciences of the Czech Republic, České Budějovice, Czech Republic

**Keywords:** Metamonada, mitochondrial carrier protein, ADP/ATP carrier, SLC25A43, mitochondrion-related organelle, mitochondrial evolution

## Abstract

Metamonada is a eukaryotic supergroup of free-living and parasitic anaerobic protists. Their characteristic feature is the presence of highly reduced mitochondria that have lost the ability to produce ATP by oxidative phosphorylation and in some cases even by substrate phosphorylation, with all ATP being imported from the cytosol. Given this striking difference in cellular ATP metabolism when compared to aerobic mitochondria, we studied the presence of mitochondrial carrier proteins (MCPs) mediating the transport of ATP across the inner mitochondrial membrane. Our bioinformatic analyses revealed remarkable reduction of MCP repertoire in Metamonada with striking loss of the major ADP/ATP carrier (AAC). Instead, nearly all species retained carriers orthologous to human SLC25A43 protein, a little-characterized MCP. Heterologous expression of metamonad SLC25A43 carriers confirmed their mitochondrial localization, and functional analysis revealed that SLC25A43 orthologues represent a distinct group of ATP transporters, which we designate as ATP-importing carriers (AIC). Together, our findings suggest that AIC facilitate the ATP import into highly reduced anaerobic mitochondria, compensating for their diminished or absent energy metabolism.

**Figure:**
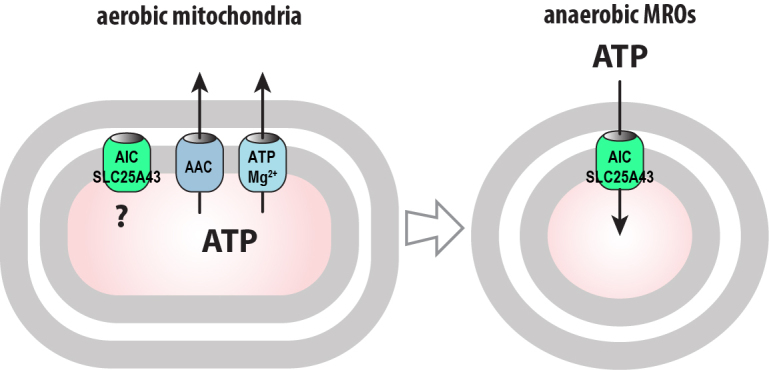

## 1. Introduction

From an evolutionary perspective, aerobically respiring mitochondria became a rich source of ATP and other metabolites, offering evolutionary benefits to eukaryotes. Nevertheless, several eukaryotic lineages have independently adapted to anoxic environments and during this process, their mitochondria lost the mitochondrial genome and capability to perform oxidative phosphorylation [1–3]. Due to their overall metabolic and morphological reduction, these organelles have been collectively named as mitochondrion-related organelles (MROs) [4].

Metamonada represent an entire supergroup of eukaryotes that have adapted to anaerobiosis either as free-living or parasitic protists [5,6]. Experimental and genomic data have revealed different types of MROs in Metamonada, ranging from the relatively complex organelles in *Anaeramoeba* [7] to the simpler hydrogenosomes of *Trichomonas vaginalis* [8] and the minimalist mitosomes of *Giardia intestinalis* [9]. An extreme adaptation has occurred in *Monocercomonoides exilis*, where the mitochondrion has been lost entirely [10]. Consistently, while some of the MROs are capable of ATP production via substrate-level phosphorylation, others, such as mitosomes, must rely on ATP transport from the cytosol to maintain processes like iron-sulfur cluster assembly or protein import [11–13].

The inner mitochondrial membrane (IMM) is impermeable to metabolites [14], and their exchange between the cytosol and the mitochondrial matrix must be facilitated by dedicated transporters, most of which are mitochondrial carrier proteins (MCP) [15]. More than 50 different MCPs function in human mitochondria, transporting a wide range of molecules such as amino and carboxylic acids, nucleotides, fatty acids and protons [16]. However, the number of MCPs varies greatly among eukaryotes and reflects the metabolic complexity of their mitochondria. In anaerobes, the loss of respiration and most catabolic pathways led to a dramatic reduction in the MCP repertoire [17,18].

MCPs are integral membrane proteins with six transmembrane α-helices that build a functional transporter in the IMM. They share a pseudo-symmetrical structure with three domains of approximately 100 amino acids in length [19]. Each domain consists of two transmembrane α-helices connected by a hydrophilic matrix loop and a small matrix α-helix [20]. The odd- and even-numbered helices contain Px[DE]xx[KR]xxxQ and [YF][DE]xx[KR] motives, respectively [21–23], that form salt-bridge networks on both sides of the membrane. An additional motif [DE]Gx_n_[YWF][KR]G at the end of matrix loops has been linked to the binding of cardiolipin [24,25].

Transport of ATP, ADP and their deoxy variants can be performed by several different MCPs, primarily the ADP/ATP carrier (AAC), which mediates equimolar exchange of ADP and ATP [26–28]. AAC is inhibited by carboxyatractyloside (CATR) and bongkrekic acid (BKA), which locks the carrier in the matrix-open or cytosol-open state, respectively [20,23]. ATP-Mg/P_i_ carriers (APCs) exchange ATP-Mg, ATP, ADP and AMP for P_i_ in an electroneutral manner [29–32]. In plants, several other carriers were shown to exchange AMP for ATP, ADP or NAD^+^ [33–36].

How the metabolic reduction of MROs has shaped the repertoire of ATP-transporting carrier proteins remains largely unknown. Specifically, has a distinct ATP carrier been required to mediate the reversed ATP flow from the cytosol into MROs? To date, ATP carriers have been identified in MROs from several anaerobes, including *Neocallimastix* sp. [37,38], *Trichomonas gallinae* [39]*, Paratrimastix pyriformis* [40] and *Antonospora locustae* [41]. Furthermore, the mitosomal carrier of *Entamoeba histolytica* has been found to exchange ATP also for 3′-phosphoadenosine-5′-phosphosulfate (PAPS) [42,43], while the *Cryptosporidium parvum* ATP carrier also transports TDP, TTP, UDP and UTP [44]. Importantly, none of these ADP/ATP exchangers could be inhibited by CATR and BKA as AACs in mitochondria of aerobes.

In this study, we analysed the diversity and evolution of MCPs in Metamonada. Through homology searches, we identified 152 metamonad MCP sequences, including 61 newly annotated MCPs missed in previous annotations. Our phylogenetic reconstruction confirmed a significant reduction in the carrier repertoire within Metamonada, highlighting the loss of AAC as the dominant ATP carrier. Instead, our data show that metamonads have adapted to rely on ATP transport via orthologues of human SLC25A43, hereafter referred to as the ATP-importing carrier (AIC). We focused on the functional characterization of three metamonad AICs. Expression in the heterologous system of *Trypanosoma brucei* indicated their localization in MROs. Furthermore, for the proteins from *Chilomastix caulleryi* and *Dysnectes brevis*, we were able to demonstrate their capability to transport ATP when expressed in the membranes of *Lactococcus lactis*. Overall, our data suggest that AIC has been preserved to mediate the reversed transport of ATP from cytosol to the MROs that lost the capacity to generate sufficient amounts of ATP for their function.

## 2. Results

### 2.1. Inventory of MCPs in the Metamonada group

**Figure 1.**
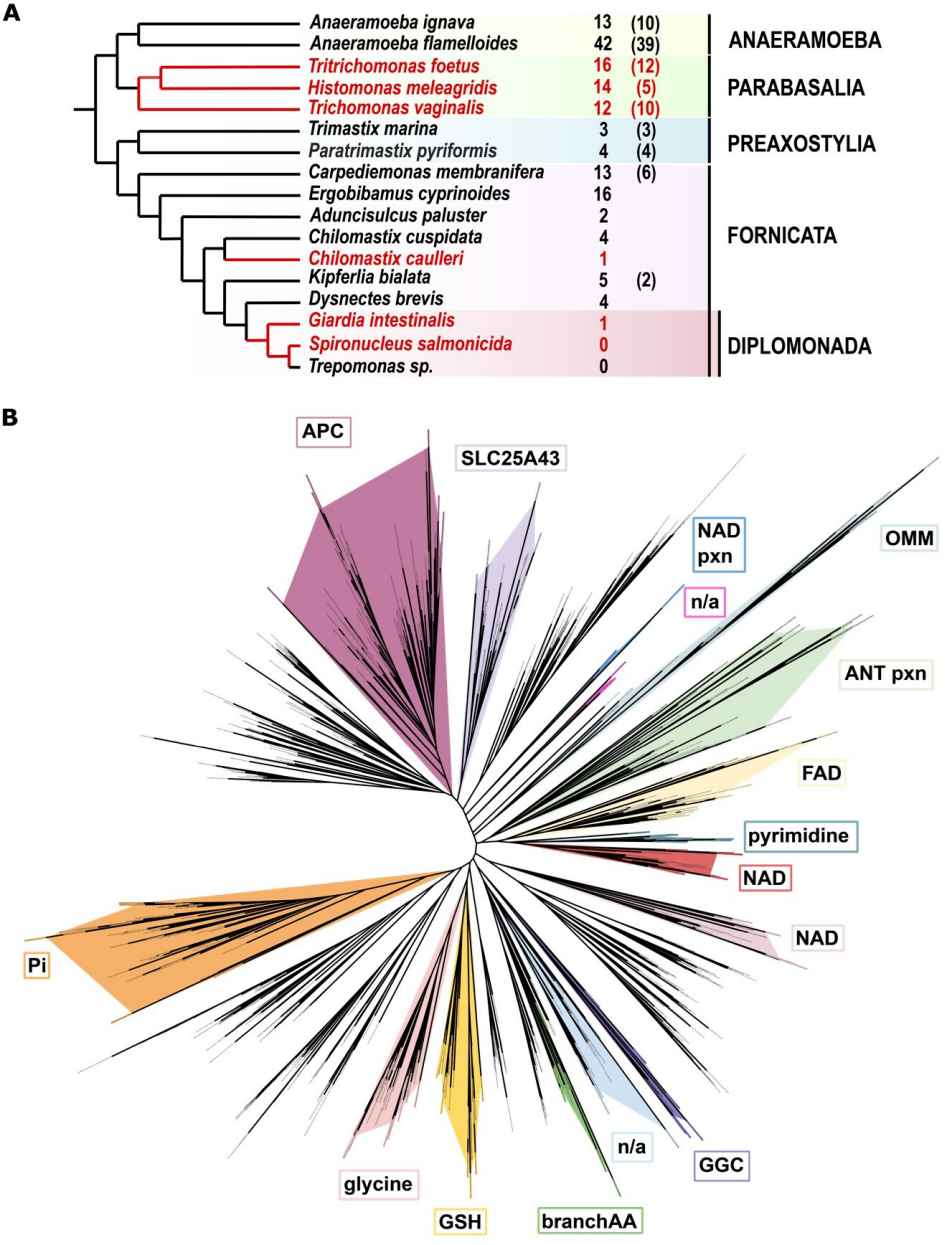
Identification of mitochondrial carrier proteins in Metamonada. (A) A cladogram of Metamonada, adapted from [47] displaying numbers of unique MCP orthologues identified in the genomic and transcriptomic data. The number of the MCPs annotated before this study is shown in brackets. Parasitic species are marked in red. (B) Phylogenetic reconstruction of 900 amino acid sequences showing the classification of Metamonada MCPs. MCPs with defined specificities from humans, yeast and *A. thaliana* were used as branch markers. Branches containing Metamonada sequences are marked in colours. APC—ATP-Mg/P_i_ carriers, ANT pxn—peroxisomal adenine nucleotide transporters, branchAA—branched amino acid carriers, GGC—GDP/GTP carriers, GSH—glutathione carriers, NAD pxn—peroxisomal NAD carriers, n/a—unknown substrate, OMM—outer mitochondrial membrane carriers.

Amino acid sequences of previously characterized MCPs from other eukaryotes were used as queries for HMM-based searches across genomic and transcriptomic datasets of 17 Metamonada species. The initial model was consecutively enriched with newly found sequences and iteratively refined until no additional sequences could be identified. Our searches retrieved 91 currently annotated MCP sequences and unveiled 60 new ones (electronic supplementary material, table S1). The most extensive repertoire, comprising 42 proteins, was identified in *Anaeramoeba flamelloides* [7], whereas only a single MCP was found in the genomes of *G. intestinalis* [45] and *C. caulleryi* [2,46]. Notably, no sequences could be identified in *Spironucleus salmonicida* and *Trepomonas sp*. by the methods used in this study (figure 1A).

### 2.2. Metamonada have orthologues of human SLC25A43

The substrate specificities of identified MCPs were inferred from the phylogenetic tree [48–52] that included well-annotated MCPs from human, yeast and *Arabidopsis thaliana*. In total, 900 amino acid sequences were used for the analysis, and the phylogenetic tree resolved 33 groups that corresponded to the substrate specificity across eukaryotic species, with Metamonada MCPs falling into 16 branches. Despite previous reports of AAC carriers in Metamonada [39,53] our analysis did not identify clear AAC protein orthologues among any Metamonada sequences. The most prevalent MCPs were the orthologues of the human SLC25A43 (figure 1B). Interestingly, the two functionally characterized ADP/ATP carriers of *T. gallinae* and *P. pyriformis* [39,40] also group here, suggesting that the SLC25A43 orthologues in Metamonada may represent a previously unidentified evolutionary group of ATP carriers as recently proposed for *P. pyriformis* [40]. Given these findings, we proposed designating them as the AIC. The single MCP orthologue found in *G. intestinalis* (GiMCP) clustered with GDP/GTP carriers (GGC), but it represented one of the most divergent sequences in the dataset to firmly predict its substrate specificity. However, it is the first MCP orthologue found in this key experimental metamonad model.

### 2.3. Sequence analysis of metamonad carriers

**Figure 2.**
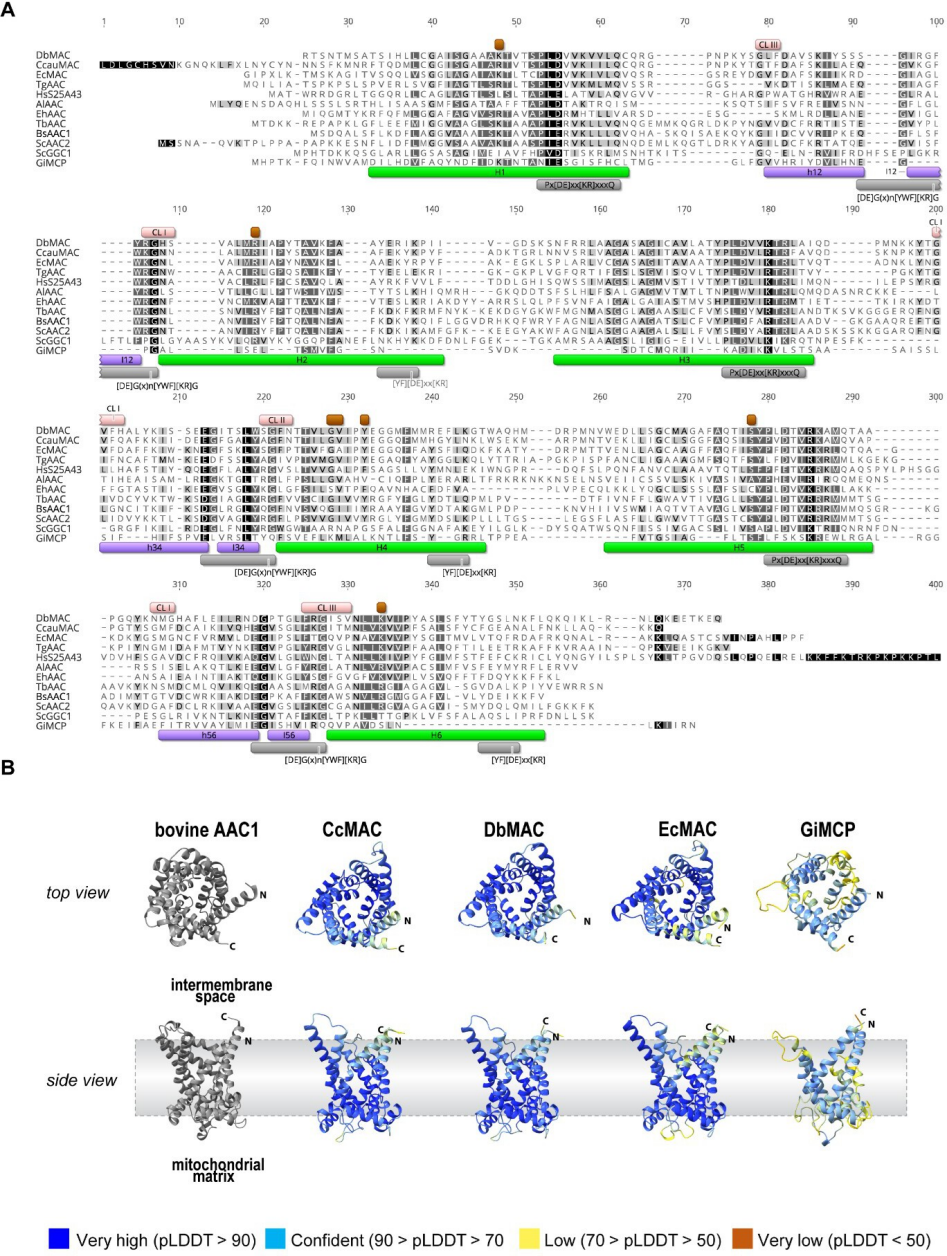
Protein sequence characterization of metamonad carriers. (A) Alignment of selected MCPs from *Chilomastix caulleryi* (CcMAC), *Dysnectes brevis* (DbMAC), *Ergobibamus cyprinoides* (EcMAC) and *Giardia intestinalis* (GiMCP) with human SLC25A43 (HsS25A43), yeast GDP/GTP carrier (ScGGC) and AACs from yeast (ScAAC2), *Bos taurus* (BsAAC1), *Antonospora locustae* (AlAAC), *Trypanosoma brucei* (TbAAC), *Entamoeba histolytica* (EhAAC) and *Trichomonas gallinae* (TgAAC). Green—transmembrane domains H1— H6; purple—helices on loops between transmembrane domains, grey—characteristic MCP motifs: Px[DE]xx[KR]xxQ on odd-numbered helices, [DE]Gx_n_[YWF][KR]G on helices in loops, and [YF][DE]xx[KR] on even numbered helices; brown—substrate binding points; pink – cardiolipin binding sites CL I—III; blue— AAC signature motif. (B) AlphaFold 3D structure predictions of Metamonada AACs along the solved structure of bovine AAC [20]. Since N- and C-termini regions of MCPs are highly disordered, the structures of MACs are missing the following residues: CcMAC 1−28 and 307−317, DbMAC 1−5 and 281−299, EcMAC 1−12 and 290−317, GiMCP 1−12. Overall pLDD scores: CcMAC 92.87, DbMAC 93.43, EcMAC 91.98 and GiMCP 80.20.

Three putative metamonad AIC orthologues from *Ergobibamus cyprinoides*, *C. caulleryi* and *D. brevis* were subjected to detailed sequence analysis. All three proteins exhibit six recognizable transmembrane domains, featuring conserved Px[DE]xx[KR]xxQ and [YF][DE]xx[KR] motifs within the odd and even numbered helices, respectively, (figure 2A) [23,25], and [DE]Gx_n_[YWF][KR]G [24,54]. This conservation translates to high accuracy (pLDDT>90) of structural predictions modelled by AlphaFold2 (figure 2B), underscoring their structural similarity to the bovine AAC structure [20] (figure 2B). In contrast, the *G. intestinalis* carrier, GiMCP, displayed negligible sequence conservation of all the important motifs (figure 2A). Still, a structure akin to the bovine AAC could be modelled by AlphaFold2, albeit with a lower pLDDT score of 80.20 (figure 2B).

Contact points in helix H4 were shown to define substrate specificity [55]. Specific G[IVLM] motif of nucleotide carriers have been defined as the interaction site with the adenine ring. While a glycine residue was found to be conserved in all three carriers, the following position is conserved only in *C. caulleryi* and *D. brevis* (figure 2A). In the analogous *E. cyprinoide*s AIC, this position is occupied by an alanine residue. Additional contact points were shown to bind the phosphate group [55] and all were found to be conserved in all three metamonad carriers (figure 2A). Finally, the selected proteins were analysed by DeepLoc2 [56] to predict their cellular localization. Mitochondrial localization was predicted for all proteins, likely due to internal targeting signals, because their sequences lack a recognizable N-terminal targeting peptide [57]. However, this is characteristic of most members of this protein family [58].

### 2.4. Mitochondrial localization of metamonad AIC carriers

**Figure 3.**
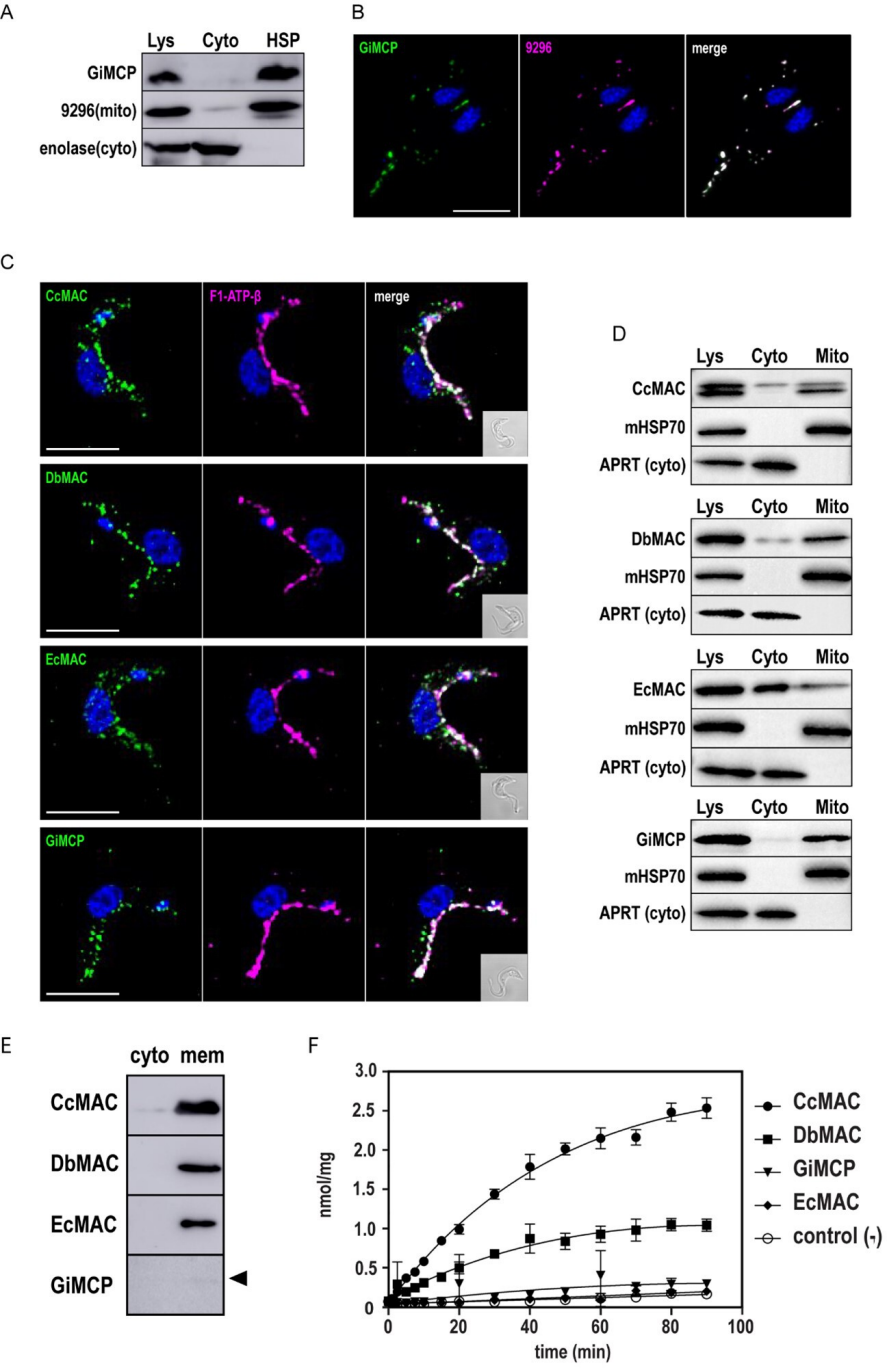
Subcellular localization of and ATP transport activity of MACs. (A) Mitochondrial localization of CcMAC, DbMAC, EcMAC and GiMCP heterologously expressed in *Trypanosoma brucei* with the C-terminal V5 tag as determined by immunofluorescence microscopy, scale bar 5 µm, F1-ATP-β—F_0_F_1_ ATPase β subunit, (B) digitonin fractionation of *T. brucei*, Lys—whole cell lysate, cyto—cytosol, mito – mitochondria, APRT—cytosolic marker adenine phosphoribosyltransferase, mHSP70—mitochondrial marker. (C). Detection of V5-tagged GiMCP in *G. intestinalis* cellular fractions, 9296—mitosomal marker, enolase— cytosolic marker. (D) Mitosomal localization of GiMCP using immunofluorescence microscopy, 9296— mitosomal marker, Scale bar 5 µm. (E) Expression of CcMAC, DbMAC, EcMAC and GiMCP in *Lactococcus lactis* as determined by the detection of Streptag in sample collected 2 h after induction at 30°C (MACs) or 25°C (GiMCP, black arrowhead). Cyto—cytosolic fraction, mem—membrane fraction. (F) [α−^33^P]ATP uptake curves of CcMAC, DbMAC, EcMAC and GiMCP measured *in vivo* in *Lactococcus lactis* cells. Strain with empty pNZ8048 plasmid served as a negative control.

The mitochondrial targeting of the three AICs was tested by expressing the genes of interest in the bloodstream form of *T. brucei,* which has served as a valuable cellular system for the analysis of proteins from non-model eukaryotes [59]. The localization of proteins with the C-terminal V5-tag was analysed using immunofluorescence microscopy (figure 3A) and western blot detection in cellular fractions (figure 3B). Mitochondrial localization was confirmed for all proteins through co-staining with the anti-β-subunit of F_O_F_1_ ATP synthase antibody (figure 3A). In the case of EcAIC, in addition to the mitochondrial presence, the signal on western blot also showed its cytosolic localization (figure 3A,B; electronic supplementary material, figure S3), which may have been due to inefficient import and subsequent protein aggregation. Likewise, mitochondrial localization was confirmed for GiMCP upon expression in *T. brucei*, underscoring the presence of mitochondrial targeting signals within metamonad carriers. Moreover, *G. intestinalis* is one of few genetically tractable systems in Metamonada. Therefore, ectopic expression of the protein with a C-terminal V5 tag and flanked by its natural 5' and 3’ UTR regions was performed in *G. intestinalis*. Western blot analysis of the cellular fraction confirmed the exclusive presence of the protein in the high-speed pellet (HSP) fraction, enriched in mitosomes (figure 3C; electronic supplementary material, figure S3). Immunofluorescence microscopy showed a specific mitosomal signal of GiMCP, as suggested by its co-localization with the mitosomal marker GL50803_9296 [60] (figure 3D). To determine whether the specific GiMCP signal could be distinguished from the outer membrane marker GiTom40, we employed expansion microscopy [61,62]. This technique allowed us to observe distinct localizations for GiTom40 and GiMCP, indicating their respective presence in the outer and inner mitosomal membranes, respectively (electronic supplementary material, figure S2).

### 2.5. AIC display ATP transport activity *in vivo*

To assess whether AICs represent functional carriers, we tested their transport activity by expressing them in the Gram-positive bacterium *L. lactis,* an excellent system for the characterization of heterologous membrane transporters [40,44,63,64]. The AIC coding sequences were codon-optimized for expression in *L. lactis* and N-terminally truncated to remove putative targeting extensions that could interfere with their membrane insertion [64] (§3). Each protein was fused to an N-terminal Strep-tag, and their expression was induced by nisin [65] (figure 3E). Of the three proteins expressed, specific ATP uptake could be successfully measured for CcAIC and DbAIC (figure 3E). However, no activity was detected for EcAIC despite its efficient expression in *L. lactis* membranes. Unfortunately, heterologous expression of GiMCP did not yield enough of the protein to assess its activity in *L. lactis* membranes (figure 3E). We attempted deleting the corresponding gene from the *G. intestinalis* genome using CRISPR/Cas9 to verify its function by reverse genetics [66]. Regardless, all attempts yielded no viable transformants, an indication of GiMCP essentiality. Finally, we tested if AICs and GiMCP could rescue the function of AAC in two other heterologous systems. First, we used an indirect assay of membrane potential determination in *T. brucei* AAC dKO. This strain is able to generate a mitochondrial membrane potential through the reverse activity of F_o_F_1_-ATP synthase [67] only when an efficient ATP carrier restores the import of ATP to mitochondria. The presence of membrane potential was followed by a fluorescent indicator safranin O [68,69] (electronic supplementary material, figure S4). The other used a knock-out strain of *Saccharomyces cerevisiae Δaac* lacking two of its AAC carriers (AAC1 and AAC2), unable to grow on non-fermentable carbon sources [70]. However, neither of these attempts proved successful (electronic supplementary material, figure S4).

### 2.6. AIC orthologues are widely present in eukaryotes

Finally, given that the identified AIC orthologues in metamonads represent ATP carriers, we decided to identify AIC orthologues also in other groups of eukaryotes. To this aim, we performed HMM-based searches in genomic and transcriptomic data available in the the comparative set (TCS) of EukProt database v3 supplemented with selected metamonad species.

**Figure 4.**
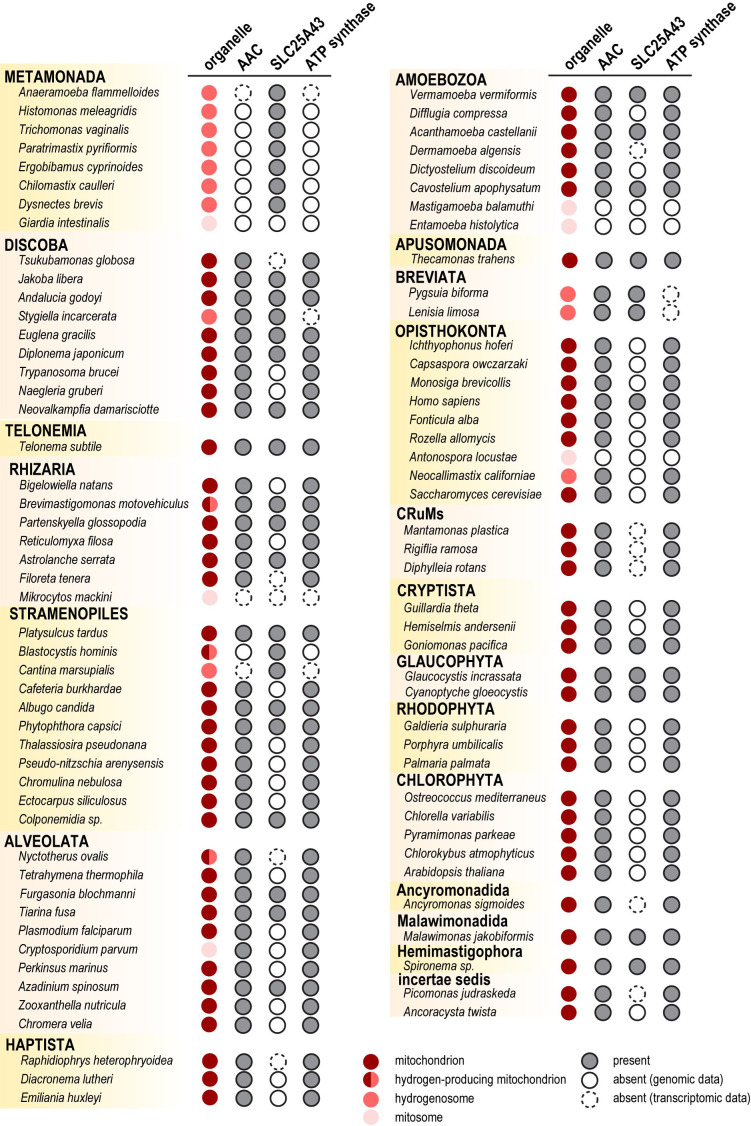
Distribution of AIC, AAC, ATP-Mg2+/Pi carrier and ATP synthase in eukaryotes. ATP synthase presence was assigned based on [47] and our HMMER-based searches in the EukProt database v3. Mitochondrion types are labelled. Eukaryotic supergroups are assigned as in [71].

This dataset of 231 species included eukaryotes with different types of mitochondrial organelles across the eukaryotic tree and with conservation or loss of AIC orthologues. Our analysis revealed the presence of AIC in 16 out of 20 eukaryotic supergroups, a total of 71 species (figure 4; electronic supplementary material, table S2). Due to the presence of AIC in metamonads that simultaneously lack AAC, ATP-Mg^2+^/Pi carrier and ATP synthase, we also mapped their occurrence in probed species. Interestingly, aerobic mitochondria with ATP synthase were almost always found to carry AAC (97% cases). The ATP-Mg²^+^/Pi carrier was largely present in the aerobes (77%) but AIC was present only in about one-fourth of the species (27%). On the contrary, AIC was never found as a sole ATP carrier when ATP synthase was present, possibly not capable of transporting large quantities of ATP. However, it only remained present as the sole ATP carrier when the ATP synthase was lost, as is typically the case in the MROs of anaerobic eukaryotes (figure 4; electronic supplementary material, table S2).

### 2.7. Discussion

The most extensively studied members of the mitochondrial carrier family are those of ADP/ATP exchange. Of the MCPs identified in Metamonada, none of the MCP grouped with the AAC, the dominant mitochondrial ADP/ATP exchanger, and only a single sequence was recognized as ATP-Mg^2+^/Pi carrier. Instead, the biochemically characterized metamonad ATP carriers [39,40] branched beside SLC25A43 (here referred to as AIC), a human member of the family with an unknown transport function. Moreover, its orthologues have been found abundantly in other metamonads suggesting that AIC orthologues represent a separate group of ATP carriers as recently proposed in the case of *P. pyriformis* [40]. This hypothesis is also supported by the ability of *D. brevis* and *C. caulleryi* MAC proteins to transport ATP, as demonstrated by our *in vivo* assay in *L. lactis* (figure 3E). However, given that neither of the MACs could complement the function of AAC proteins in *S. cerevisiae* and *T. brucei* mitochondria, it is likely that both proteins are not functionally redundant.

One of the possibilities is that compared to AAC, AIC has very different bioenergetic properties and is better suited for mitochondria with no functional ATP synthase complex that would harness the proton motive force generated by a respiratory chain.

Potentially, there could be further aspects of SLC25A43/AIC function, as the expression of the protein was found to be silenced or its gene damaged in different cancer cell lines [72,73]. The experimental silencing of SLC25A43/AIC affected the cell cycle progression of different cancer cell lines as well as their response to cytostatic drugs [74,75]

**Figure 5.**
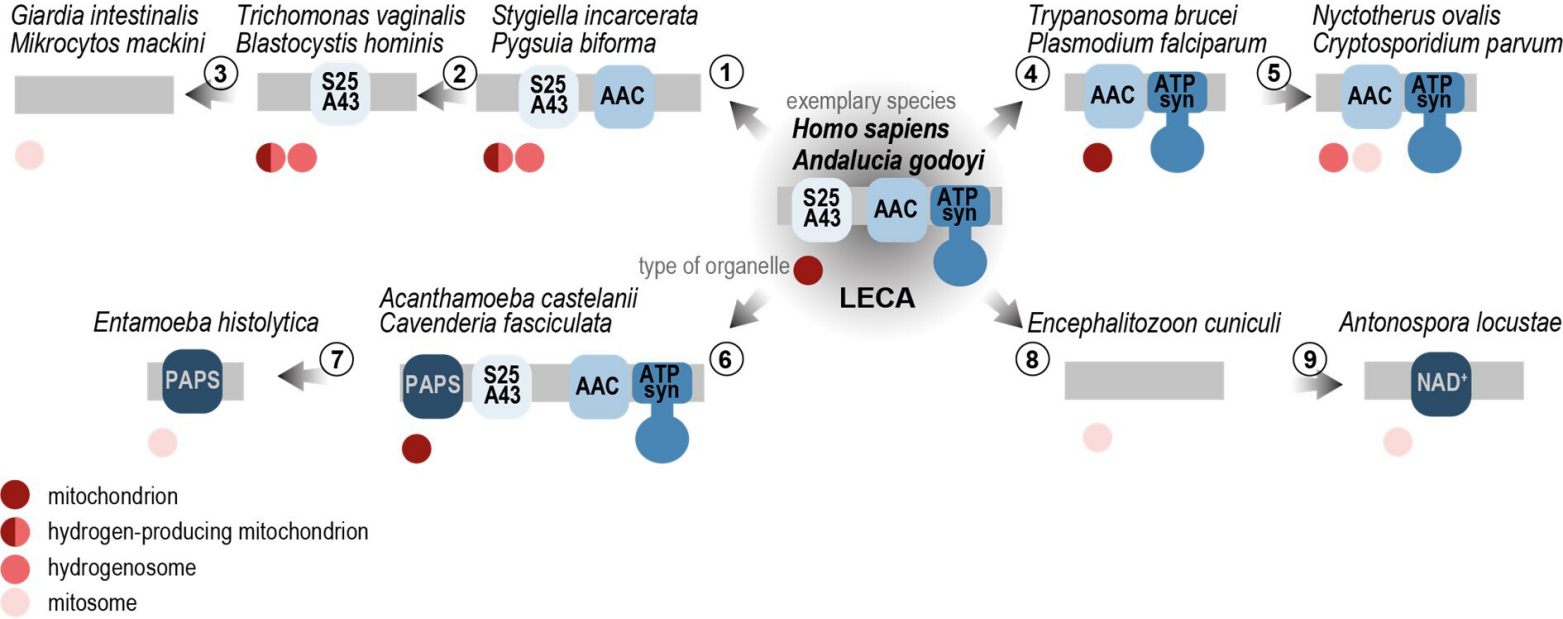
Evolutionary history of AIC, AAC and ATP synthase in mitochondria and MROs. The ancestral arrangement of AIC, AAC and ATP synthase as it was present in the last eukaryotic common ancestor (LECA) [77] and can be found in various species across the eukaryotic tree, here exemplified by *Andalucia godoyi* and *Homo sapiens*. In some eukaryotes, the mitochondrial adaptation to anoxic environment led to the loss of ATP synthase (1) but preservation of AAC and AIC. This arrangement is observed in MROs such as those of *Stygiella incarcerata* and *Pygsuia biforma*. Only when ATP synthase is lost AAC becomes redundant and only AIC can be retained as ATP transporter, as is the case for several MROs including *Trichomonas vaginalis* and *Blastocystis hominis*. Finally, in the most reduced MROs known as mitosomes, neither of these components are present, as in *Giardia intestinalis* or *Mikrocytos mackini* (3). In some aerobic eukaryotes, AIC was lost while ATP synthase and AAC remained present (4). This arrangement remained also in reduced MROs of anaerobic eukaryotes (5) such as *Nyctotherus ovalis* or *Cryptosporidium parvum*. On the other hand, alternative carriers that can transport ATP were acquired by horizontal gene transfer, e.g. amoebozoans such as *Acanthamoeba castellani* and *Cavenderia fasciculata* gained a PAPS/PAP carrier (6), which remained a sole carrier capable transporting ATP in mitosomes of *Entamoeba histolytica* (6). Microsporidia as obligatory intracellular parasites lost all carrier proteins (8) and replaced them by non-homologous bacterial proteins [78,79]. However, a single carrier closely related to a locust NAD^+^ transporter is uniquely present in the mitosomes of *Antonospora locustae* (9)*,* a locust parasite. MRO types and ATP synthase presence were assigned as in figure 4. AAC—ADP/ATP carrier orthologue, ATP syn—ATP synthase (complex V), NAD^+^—NAD^+^ carrier, PAPS—3′-phosphoadenosine−5′-phosphosulfate/3'-phosphoadenosine 5'-phosphate carrier, AIC—ATP import carrier.

Importantly, a recent screening identified SLC25A43/AIC as one of the main targets of cellular oxidative toxicity, since its experimental removal was associated with an increased resistance to oxidative stress [76]. Hence, it is possible that in anoxic environments, mitochondrial organelles such as MROs found in Metamonada rely on AIC without the risk of increased oxidative stress. The search for orthologues of AIC across the eukaryotic tree (figure 4) indicated their widespread presence among eukaryotes, with sporadic losses in various lineages. Alongside this, a pattern of the distribution of ATP synthase, AAC and AIC may be observed, suggesting that ATP synthase may have required the presence of the AAC, whereas the loss of these components is often accompanied by the conservation of AIC. Hence, we hypothesize that AIC was a pre-requisite for AAC and ATP synthase loss and that it likely took place through a gradual process (figure 5). The ancestral arrangement consists of AIC, AAC and ATP synthase and is common in various species (e.g. *Andalucia godoyi* and *Homo sapiens*). The primary step of reduction was probably the loss of ATP synthase. Such arrangement of co-existing AIC and AAC without ATP synthase is observed in the hydrogenosomes of jakobid *Stygiella incarcerata* and breviates *Pygsuia biforma* and *Lenisia limosa*. Once the ATP synthase was lost, AAC may have become dispensable as well. The unaccompanied AIC can be found in anaerobic MROs of many species carrying either hydrogen-producing mitochondria or hydrogenosomes, such as several metamonads and stramenopile *Blastocystis hominis*. Finally, none of these components may be present, as is the case with highly reduced mitosomes of *G. intestinalis* or *Mikrocytos mackini* of the Rhizaria group.

In contrast, many eukaryotes show that AIC was lost as the first of the trio. This includes some opisthokonts, plants and protist species such as *T. brucei* or *Plasmodium falciparum* (figure 5). This arrangement can also lead to the reduction of mitochondria to MRO, yet both AAC and ATP synthase have been retained such as in the case of alveolates *Nyctoterus ovalis* and *C. parvum*.

Due to this observation and the unsuccessful attempts to complementation the phenotype of *Δaac* mutant strains (electronic supplementary material, figure S4), we conclude that ATP synthase requires a potent ADP/ATP carrier and cannot be replaced by AIC. Only after the loss of ATP synthase does AAC become redundant, which further highlights the metabolic ties between AAC and ATP synthase [26].

However, there are also rare cases when the ATP transport is mediated by an alternative carrier that was acquired independent of AIC. An amoebozoan anaerobe *E. histolytica* relies on a PAPS/PAP carrier, which acts as the sole ATP transporter in its mitosomes [42,43]. In this case, this was likely possible due to the ancestral acquisition of the PAPS/PAP carrier in the Amoeboza group, as evidenced by its occurrence in related aerobic species *Acanthamoeba castellani* and *Cavenderia fasciculata* (electronic supplementary material, figure S5A). Similarly, Microsporidia, obligatory intracellular parasites, lost all MCPs [80] and replaced them by non-homologous bacterial proteins [78,79]. Yet, a single ATP carrier was found in the mitosomes of *Antonospora locustae* [41]. The proteins are related to NAD^+^ transporters and are almost identical (99% similarity) to a MCP from its host, desert locust *Schistocerca gregaria* (electronic supplementary material, figure S5B), strongly suggesting a recent horizontal gene transfer from the host to the parasite, and a secondary acquisition of an mitochondrial ATP carrier protein.

Throughout the eukaryotic tree of life, the number of carriers per organism depends on the set of metabolites exchanged across IMM. The only carrier identified in the genome of *G. intestinalis* (GiMCP) represents one of the most divergent proteins identified in our dataset, which is probably why it escaped previous bioinformatic analyses [60,81–83]. Given the degree of divergence of GiMCP and the absence of identifiable MCP in other related species belonging to the Diplomonada group, this supports the evolutionary divergence of this group of eukaryotes [45,84,85]. While we were able to show its mitosomal localization, its function as an ATP carrier could not be resolved. Thus, further experiments are required to test the substrate specificity of this carrier, which could also shed light on the energetics of the organelle, which is entirely devoted to the formation of Fe-S clusters [12].

Apart from these rather sporadic cases of intriguing cellular adaptations, our work proposes that, fixation of AIC is an evolutionary-favoured eukaryotic adaptation to anaerobic conditions, occurring together with the reduction in energy metabolism/ATP production in MROs across the eukaryotic tree.

## 3. Material and methods

### 3.1. Identification and sequence analysis of putative MCP sequences

The candidates for MCPs were searched using the HMMER version 3.3.2 (http://hmmer.org/) in the EukProt database version 3 [46], transcriptomes of *Carpediemonas*-like organisms (CLOs) [2] and *Anaeramoeba* species [7], in genomes and transcriptomes in databases GiardiaDB (https://giardiadb.org) and TrichDB (https://trichdb.org), in *Histomonas meleagridis* genome [86] and transcriptome [87] and *P. pyriformis* conceptual proteome [88]. Next, reciprocal BLASTP (https://blast.ncbi.nlm.nih.gov) and HHpred [89,90] searches were performed.

For the phylogenetic analysis, the dataset was enriched with selected proteins from InterPro family IPR002067 [91] and with annotated sequences from human, yeast and *A. thaliana* that served as markers for the individual branches. To remove duplicates or highly similar sequences, the initial dataset was clustered with MMseqs2 [92] using the easy-cluster algorithm with a minimal sequence identity threshold of 0.8 and minimum alignment coverage of 0.8. Multiple sequence alignment was created in MAFFT version 7 [93]. The E-INS-i algorithm with default parameters was used. Subsequently, poorly aligned regions were automatically removed by trimAl [94] with the gappyout mode. Phylogenetic analysis was performed with IQ-TREE2 software [95]. The best-fitting model was obtained with the ModelFinder [96]. To reduce the computation time, the UFBoot (ultra-fast bootstrap) method was employed [97]. The representative alignment of putative Metamonada and reference MCPs was done in Geneious Prime 2023.1.1 (https://www.geneious.com). 3D structures of Metamonada AACs were predicted with the AlphaFold2 software [98], using the online ColabFold interface with default parameters [99,99]. For *G. intestinalis* GL50803_17286 the search was supplemented with a custom MSA.

### 3.2. Plasmid construction and cloning

For the episomal expression in *G. intestinalis* GL50803_17286 gene with 5’ and 3’UTR regions were amplified by PCR from genomic DNA and cloned into a pTG vector [60] with C-terminal V5-tag. For the heterologous expression in *T. brucei* GL50803_17286 the gene was amplified by PCR from genomic DNA. *Chilomastix caulleri* ORF 424, *D. brevis* ORF 6817, and *E. cyprinoides* ORF 2013 were *T. brucei* codon optimized and obtained from GenScript. All genes were amplified by PCR and cloned into tetracycline-inducible vector pT7_v5 [100] with a C-terminal V5-tag. For the expression in *L. lactis*, all genes were N-terminally truncated [64]: *G. intestinalis* GL50803_17286 (7-229), *C. caulleri* ORF 424 (31-317), *D. brevis* ORF 6817 (8-299) and *E. cyprinoides* ORF 2013 (15-317). All genes with N-terminal Strep-tag were L. *lactis* codon optimized and obtained from GenScript. All genes were cloned directly into nisin-inducible vector pNZ8048 [63] via restriction digest and ligation using NcoI and KpnI sites.

### 3.3. Culture and transfection

*G . intestinalis* trophozoites ATCC 50803 strain/WB clone C6 were cultured in TYI-S-33 medium supplemented with 10% (v/v) heat-inactivated ABS and 0.1% (v/v) bovine bile at 37°C. Cells were electroporated as previously described [101]. Briefly, 10^7^ cells were harvested by centrifugation at 1200 × g at 4°C for 10 min and resuspended in 300 μl of media. 50 μg of circular plasmid were electroporated (350 V, 1000 μF, 750 Ω) into the cells using 4 mm electroporation cuvettes on a Bio-Rad Gene Pulser set to the exponential protocol. Cells were selected with 57 μg ml^−1^ of puromycin.

The bloodstream form *T. brucei* Lister 427 strain and the AAC double knockout (dKO) [67] were cultured in HMI-11 media supplemented with 10% (v/v) FBS at 37°C with 5% (v/v) CO_2_ in the presence of 2.5 μg ml^−1^ of G418 and 25 μg ml^−1^ of hygromycin. 0.8 × 10^6^ dKO trypanosomes were electroporated with 20 μg of NotI-linearized plasmid using human T cell nucleofector solution (Lonza) and Nucleofector 2b (Lonza). Transfectants were selected with 0.1 μg ml^−1^ of puromycin. Expression of V5-tagged proteins was induced by tetracycline (100 ng ml^−1^) for 24 h.

*L. lactis* strain NZ9000 was cultured in M17 media (Formedia) supplemented with 0.5% (w/v) glucose at 30°C. Preparation of electrocompetent bacteria and transformation were done as described previously [63]. Transformed cells were selected with 10 μg ml^−1^ of chloramphenicol. The expression of Strep-tagged proteins was induced at OD600 0.4–0.5 by 5 ng ml^−1^ nisin for 6 h at 30°C or 25°C for the expression test. For transport assay cells were induced for 1 h at 30°C (Chilomastix *caulleri* ORF 424, *D. brevis* ORF 6817 and *E. cyprinoides* ORF 2013) or 5 h at 25°C (GL50803_17286).

### 3.4. Cells fractionation and western blot analysis

*G. intestinalis* cell fractions were prepared as previously described [102]. Briefly, 5 × 10^9^ cells were harvested by centrifugation at 1200 × g at 4°C for 15 min and washed with ice-cold PBS. The pellet was resuspended in ST buffer (10 mM Tris-HCl pH 7.4, 0.5 mM KCl, 250 mM sucrose) supplemented with protease inhibitors (cOmplete, EDTA-free; Roche). Cells were disrupted on ice by sonication (1 s on/1 s off pulse, amplitude 40% for 6 min), a sample was collected for Western blot (whole cell lysate fraction) and the rest of the lysate was centrifuged at 2680 × g at 4°C for 20 min. The supernatant was then centrifuged at 180 000 × g at 4°C for 30 min. The resulting pellet and supernatant were collected for western blot analysis (HSP and cytosolic fraction, respectively) using primary antibodies: rat anti-enolase polyclonal antibody at dilution 1 : 2000, rabbit anti-GL50803_9296 polyclonal antibody at dilution 1 : 2000 and rat anti-V5 epitope (V5 tag) monoclonal antibody at dilution 1 : 1000 and secondary antibodies: HRP-conjugated goat anti-rabbit and HRP-conjugated goat anti-rat monoclonal IgG antibodies at dilution 1 : 2000.

*T. brucei* digitonin subcellular fractionation was done as previously described [103]. Briefly, 1 × 10^8^ cells were harvested by centrifugation at 1300 × g at 4°C for 10 min and washed with PBS-G. A sample was collected for western blot (whole cell lysate fraction). The rest of the pellet was resuspended in SoTE buffer (20 mM Tris-HCl pH 7.5, 0.6 M sorbitol, 0.2 mM EDTA) followed by the addition of SoTE containing 0.03% (w/v) digitonin and incubated on ice for 5 min. The sample was centrifuged at 6800 × g at 4°C for 3 min. The resulting pellet and supernatant (mitochondrial and cytosolic fractions, respectively) were collected for western blot analysis using primary antibodies: rabbit anti-APRT [104] polyclonal antibody at dilution 1 : 200, rabbit anti-F_o_F_1_ ATP synthase β subunit polyclonal antibody at dilution 1 : 2000 [105], mouse anti-V5 epitope (V5 tag) monoclonal IgG antibody at dilution 1 : 2000 and secondary antibodies: HRP-conjugated goat anti-rabbit and HRP-conjugated goat anti-mouse monoclonal IgG antibodies at dilution 1 : 2000.

### 3.5. Immunofluorescence and expansion microscopy

*G. intestinalis* cells were fixed and immunolabelled as previously described [106] Primary antibodies used were rabbit anti-GL50803_9296 polyclonal antibody [60] at dilution 1 : 1000 and anti-V5 epitope (V5 tag) rat monoclonal antibody at dilution 1 : 500. Secondary antibodies used were Alexa Fluor 594-conjugated goat anti-rabbit monoclonal IgG antibody at dilution 1 : 1000 and Alexa Fluor 488-conjugated goat anti-rat monoclonal IgG antibody at dilution 1 : 1000. Slides were mounted with Vectashield containing DAPI. Images were taken with a Leica TCS SP8 WLL SMD-FLIM inverted confocal microscope. For expansion microscopy *G. intestinalis* cells were fixed and immunolabelled as previously described [61]. Primary antibodies used were rabbit anti-Tom40 polyclonal antibody [107] at dilution 1 : 1000 and anti-V5 epitope (V5 tag) rat monoclonal antibody at dilution 1 : 1000. Secondary antibodies used were Alexa Fluor 594-conjugated goat anti-rabbit monoclonal IgG antibody at dilution 1 : 500 and Alexa Fluor 488-conjugated goat anti-rat monoclonal IgG antibody at dilution 1 : 500. Images were taken with Nikon CSU-W1 spinning disk confocal microscope.

*T. brucei* cells were harvested and fixed with 3.7% (w/v) paraformaldehyde in PBS for 10 min at room temperature. The cell suspension was then applied to the poly-lysine-coated coverslip and permeabilized with 0.1% (v/v) Triton-X in PBS for 10 min at room temperature. Primary antibodies used were rabbit anti-F_0_F_1_ ATPase β subunit polyclonal antibody at dilution 1 : 500 [105] and anti-V5 tag rat monoclonal antibody at dilution 1 : 1000. Secondary antibodies used were Alexa Fluor 594-conjugated goat anti-rabbit monoclonal IgG antibody at dilution 1 : 1000 and Alexa Fluor 488-conjugated goat anti-rat monoclonal IgG antibody at dilution 1 : 1000. Slides were mounted with Vectashield containing DAPI. Images were taken with a Leica TCS SP8 WLL SMD-FLIM inverted confocal microscope.

All image processing and analysis were performed in Huygens Professional version 22.10 (Scientific Volume Imaging) and ImageJ/Fiji software [108].

### 3.6. Uptake of radioactively labelled ATP into *Lactococcus lactis*

Uptake experiments were carried out as previously described [63], modified. Briefly, 1 h after nisin induction cells were harvested by centrifugation at 5000 × g at 4°C for 10 min and were washed with ice-cold PIPES buffer (10 mM PIPES, 50 mM NaCl). Bacteria were incubated in PIPES buffer with 10 µM [α−^33^P]ATP (0.5 µCi ml^−1^) at room temperature for indicated periods. The uptake of ATP was stopped by the addition of an ice-cold PIPES buffer. Cells were vacuum filtered through 0.45 µm cellulose nitrate membrane filters (Whatman) and washed once with ice-cold PIPES buffer. The retained radioactivity was quantified in Filter-Count (PerkinElmer) scintillation counting cocktail in a Triathler Multilabel Tester gamma counter (Hidex). Data processing and graph generation were performed in GraphPad Prism (Dotmatics).

## Data Availability

All data are part of the manuscript and the supplementary data files. Supplementary material is available online [109].
